# Isolation, identification and characterization of *Paenibacillus polymyxa* CR1 with potentials for biopesticide, biofertilization, biomass degradation and biofuel production

**DOI:** 10.1186/s12866-016-0860-y

**Published:** 2016-10-18

**Authors:** Brian Weselowski, Naeem Nathoo, Alexander William Eastman, Jacqueline MacDonald, Ze-Chun Yuan

**Affiliations:** 1 London Research and Development Centre, Agriculture & Agri-Food Canada, 1391 Sandford Street, London, ON N5V 4T3 Canada; 2 Department of Biology, Biological and Geological Sciences Building, University of Western Ontario, London, ON N6A 5B7 Canada; 3 Department of Microbiology & Immunology, Dental Science Building Rm. 3014, University of Western Ontario, London, ON N6A 5C1 Canada

**Keywords:** *Paenibacillus polymyxa*, plant growth promotion, PGPR, antagonism, biocontrol, biopesticide, biological nitrogen fixation, diazotroph, biofertilizer, biomass degradation

## Abstract

**Background:**

*Paenibacillus polymyxa* is a plant-growth promoting rhizobacterium that could be exploited as an environmentally friendlier alternative to chemical fertilizers and pesticides. Various strains have been isolated that can benefit agriculture through antimicrobial activity, nitrogen fixation, phosphate solubilization, plant hormone production, or lignocellulose degradation. However, no single strain has yet been identified in which all of these advantageous traits have been confirmed.

**Results:**

*P. polymyxa* CR1 was isolated from degrading corn roots from southern Ontario, Canada. It was shown to possess in vitro antagonistic activities against the common plant pathogens *Phytophthora sojae* P6497 (oomycete), *Rhizoctonia solani* 1809 (basidiomycete fungus), *Cylindrocarpon destructans* 2062 (ascomycete fungus), *Pseudomonas syringae* DC3000 (bacterium), and *Xanthomonas campestris* 93-1 (bacterium), as well as *Bacillus cereus* (bacterium), an agent of food-borne illness. *P. polymyxa* CR1 enhanced growth of maize, potato, cucumber, *Arabidopsis*, and tomato plants; utilized atmospheric nitrogen and insoluble phosphorus; produced the phytohormone indole-3-acetic acid (IAA); and degraded and utilized the major components of lignocellulose (lignin, cellulose, and hemicellulose).

**Conclusions:**

*P. polymyxa* CR1 has multiple beneficial traits that are relevant to sustainable agriculture and the bio-economy. This strain could be developed for field application in order to control pathogens, promote plant growth, and degrade crop residues after harvest.

## Background

Plant-growth promoting rhizobacteria can be exploited in agriculture as an environmentally friendlier alternative to chemical fertilizers and pesticides, which often pollute the environment [1–3]. Such bacteria naturally inhabit plant roots and the surrounding soil (rhizosphere), where they utilize plant-derived nutrients while benefiting the plant through a variety of direct or indirect mechanisms [4]. Direct mechanisms can include fixing atmospheric nitrogen, solubilizing mineral phosphates [5–7], synthesizing phytohormones such as indole-3-acetic acid (IAA) that are readily taken up by plant roots, and enhancing plant tolerance to abiotic stress through lowering host ethylene levels by 1-aminocyclopropane-1-carboxylate (ACC) deaminase activity [8–11]. Indirect mechanisms of plant-growth promotion include inhibition of phytopathogens, induction of plant systemic resistance against pathogens, stabilization of soil aggregates, and maintenance of soil nutrients and structure [12].

The plant-growth promoting rhizobacterium *Paenibacillus polymyxa* has attracted considerable attention because of the demonstrated abilities of its various strains to encourage crop growth via one or more mechanisms, as well as produce lignocellulose-modifying enzymes [13] which could contribute to in situ degradation of crop residues that are often burnt, affecting air quality and public health [14]. Among the most studied strains, *P. polymyxa* SQR-21 and E681 are known primarily for their ability to suppress plant disease [15, 16], while *P. polymyxa* P2b-2R promotes plant growth by fixing nitrogen [17, 18].

Comparative genomic analyses of multiple strains reveal that *P. polymyxa* gene clusters encoding antimicrobial peptides are structurally and functionally diverse, perhaps having been acquired by horizontal transfer from other species [19, 20]. Genes that encode enzymes involved in lignocellulose degradation are also somewhat variable among strains [20], and while all studied strains have putative genes for phosphate solubilization and production of the plant hormone IAA, only a few contain the *nif* gene cluster required for nitrogen fixation [19, 20]. To our knowledge, no single *P. polymyxa* strain has yet been described in which all five of these advantageous traits have been confirmed in vivo (antimicrobial, lignocellulose-degrading, phosphate solubilizing, IAA-producing, nitrogen fixing).

While the recently sequenced genome of *P. polymyxa* CR1 suggests that this strain has all of the mentioned traits [20], studies have not yet been published confirming these abilities. The current work therefore describes the isolation of *P. polymyxa* CR1 from degrading corn roots, its in vitro antagonistic activities against common plant pathogens, and its ability to promote growth of important agricultural crops. We further confirm the capability of this strain to fix atmospheric nitrogen fixation, utilize inorganic phosphate, produce the plant hormone IAA, and degrade the major components of lignocellulose.

## Results and discussion

### Species identification

**Fig. 1:**
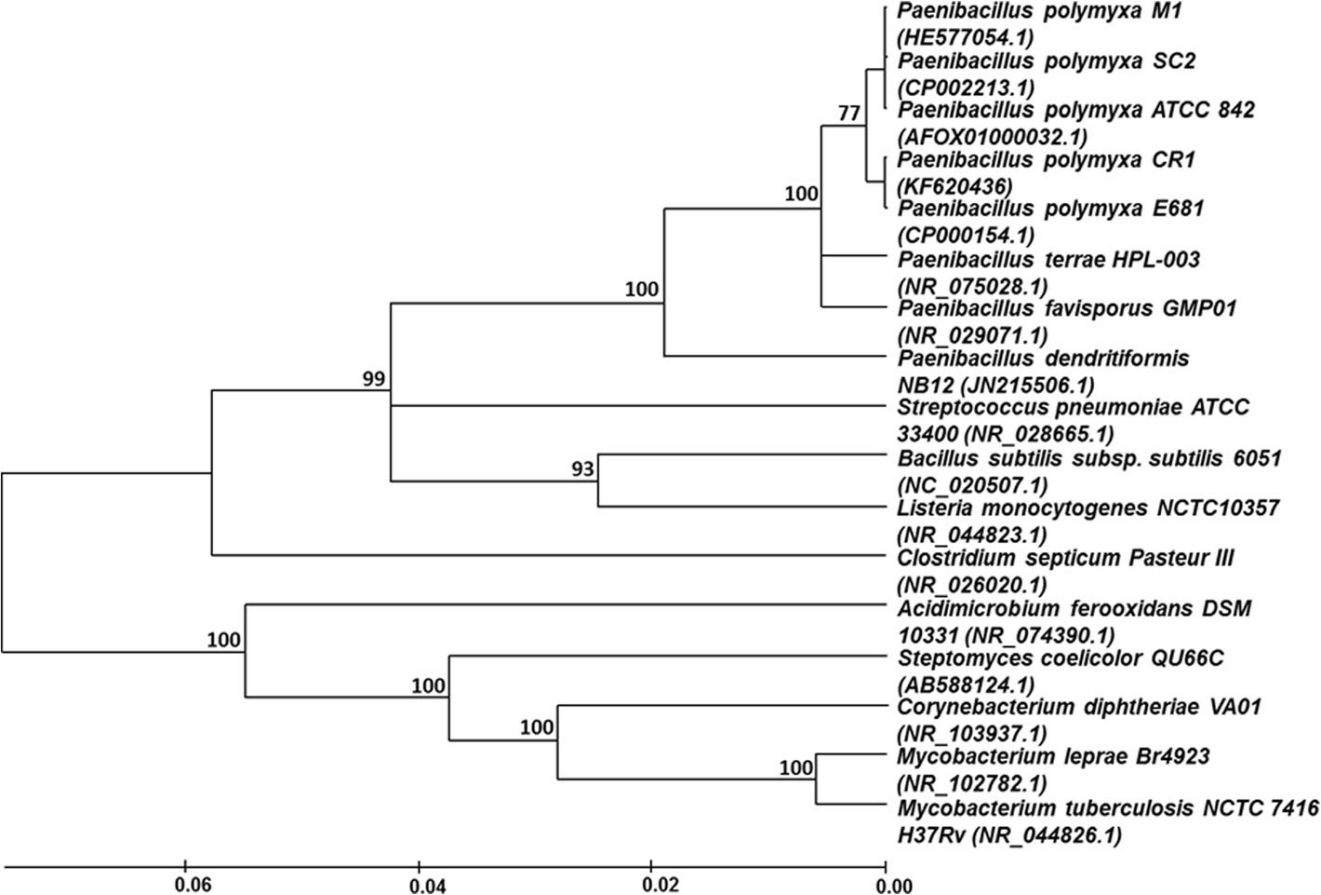
Phylogenetic tree of bacterial 16S rRNA sequences revealing *P. polymyxa* evolutionary divergence. Manually refined 16S rRNA sequences were aligned using MEGA 6 and the tree was constructed using the neighbor-joining method (approximately 1400 bp length fragments after refinement). Bootstrap values are indicated at tree branching points. *P. polymyxa* strains CR1 and E681, in reference to all *P. polymyxa* strains, are considered to be monophyletic, while remaining closely related to M1, SC2 and ATCC 842 strains

*P. polymyxa* strain CR1 was isolated from degrading corn roots and named based on phylogenetic analysis and phenotypic characterization, where CR1 is for “corn rhizobacterium 1”. *P. polyxyxa* CR1 formed hard, sticky colonies when grown on 1/5 NPT agar, exhibiting a translucent to white-yellow color. Phylogenetic analysis was based on the 16S rRNA gene sequenced in this work and deposited in Genbank (accession no. KF620436.1, the isolate’s full genome has since been sequenced [20–22]). The 16S rRNA gene was aligned against the NCBI nucleotide database using Blastn, showing 99 % identity to sequences from *P. polymyxa* strains E681 (CP000154.1), SC2, and M1. Identity to sequences from other *Paenibacillus* species was lower, e.g. 94 % identity to *Paenibacillus validus* (AB073203), 93 % to *Paenibacillus koreensis* (AF130254), and 93 % to *Paenibacillus larvae* (X60619). These 16S rRNA gene sequences, along with those from other relevant bacteria, were used to construct a phylogenetic tree (Fig. 1).

### Antagonistic activity against microbial pathogens

**Fig. 2:**
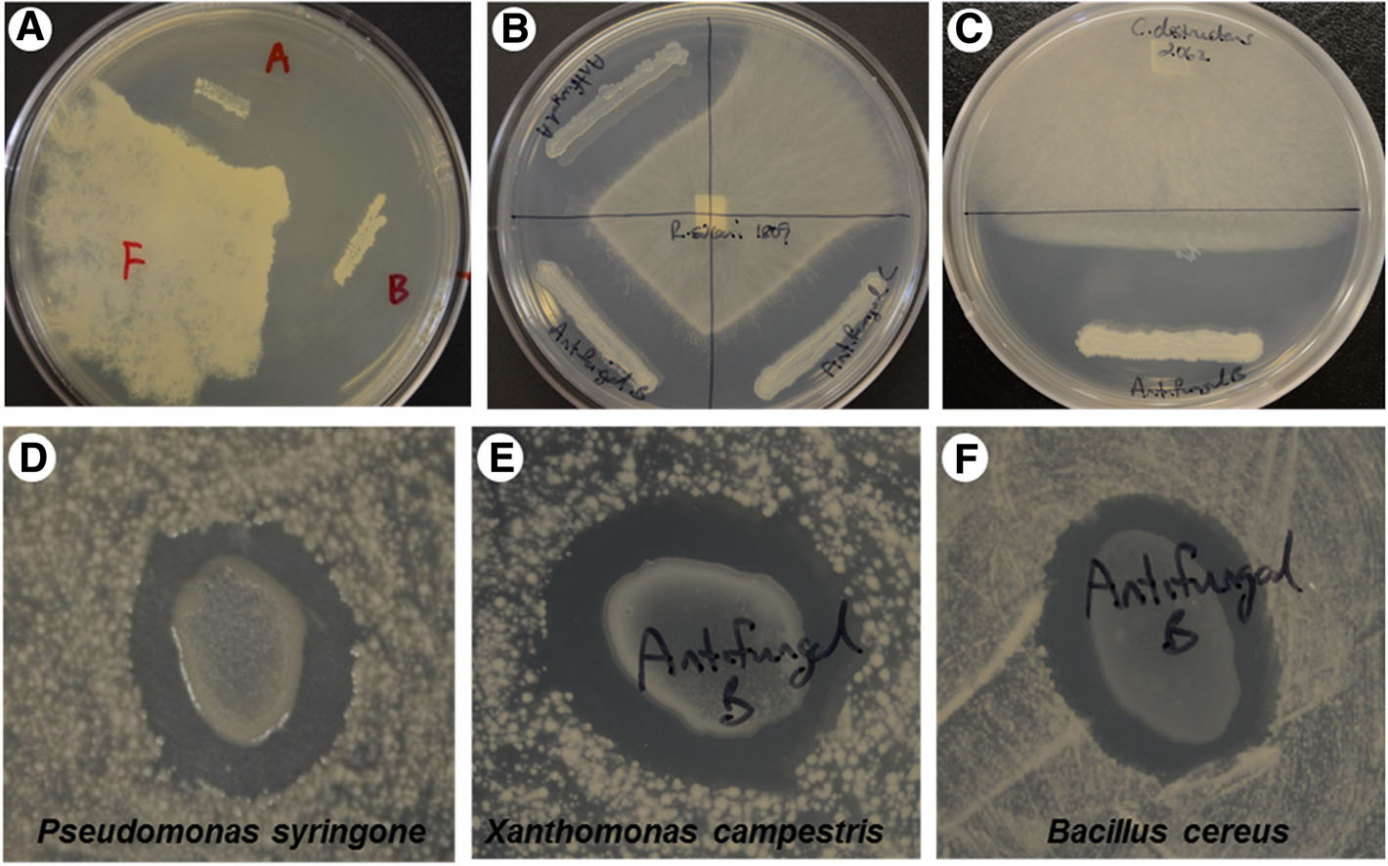
in vitro antagonistic activity of *P. polymyxa* CR1 against selected pathogens. Dual culture assay tests show zones of inhibition which indicate antagonistic activity against **a** *Phytopthora sojae* P6497; **b** *Rhizoctonia solani* 1809; **c** *Cyindrocarpon destructans* 2062; **d** *Pseudomonas syringone* DC3000; **e** *Xanthomonas campestris* 93-1; **f** *Bacillus cereus* BcMOR28. Tests were done in triplicate, representative plates are shown

A total of 1293 bacterial colonies were isolated from degrading corn roots and further screened for antagonism against pathogenic microorganisms using a dual-culture technique. Of the tested isolates, 12 demonstrated growth antagonism towards at least one phytopathogen. *P. polyxyxa* CR1, referred to as “antifungal B” prior to species identification, grew rapidly compared to the other eleven isolates (data not shown) and exhibited antagonism toward six tested pathogens (Fig. 2): *Phytophthora sojae* P6497 (an oomycete causing stem and root rot of soybean, Fig. 2a), *Rhizoctonia solani* 1809 (a basidiomycete fungus pathogen of soybean, Fig. 2b), *Cylindrocarpon destructans* 2062 (an ascomycete fungus causing root rot of ginseng, Fig. 2c); and the bacteria *Pseudomonas syringae* DC3000 (bacterial speck, Fig. 2d), *Xanthomonas campestris* 93-1 (bacterial blight, cankers and leaf spots, Fig. 2e), and *Bacillus cereus* BcMOR28 (causes human foodborne illness, Fig. 2f). It did not affect growth of *Agrobacterium tumefaciens*, the causal agent of crown gall disease (data not shown). These pathogens were tested because of their phylogenetic diversity, relevance, and availability in our lab. The observations suggest that *P. polyxyxa* CR1 produces considerable amounts of anti-microbial substances that antagonize the growth of a broad range of microbial pathogens.

Various strains of *P. polymyxa* have previously been found to antagonize microorganisms related to some of the ones tested here, including *Phytophthora* species [23, 24], *R. solani* [25, 26], *P. syringae* [27], and *X. campestris* [28]. In some cases, responsible antimicrobial compounds have been identified: fusaricidin against *Phytophthora capsici* [29], a ~35 kDa antifungal peptide against *R. solani* [25, 26], and gavaserin and saltavalin against *X. campestris* [28]. Notably, not all strains of *P. polymyxa* are antagonistic toward these organisms [24], an observation which is consistent with the diversity among strains in their gene clusters that encode antimicrobial peptides [19, 20]. The specific antimicrobial compounds produced by *P. polymyxa* CR1 are yet to be identified.

### Plant growth promotion (corn, cucumber, tomato, potato and *Arabidopsis*)

**Fig. 3:**
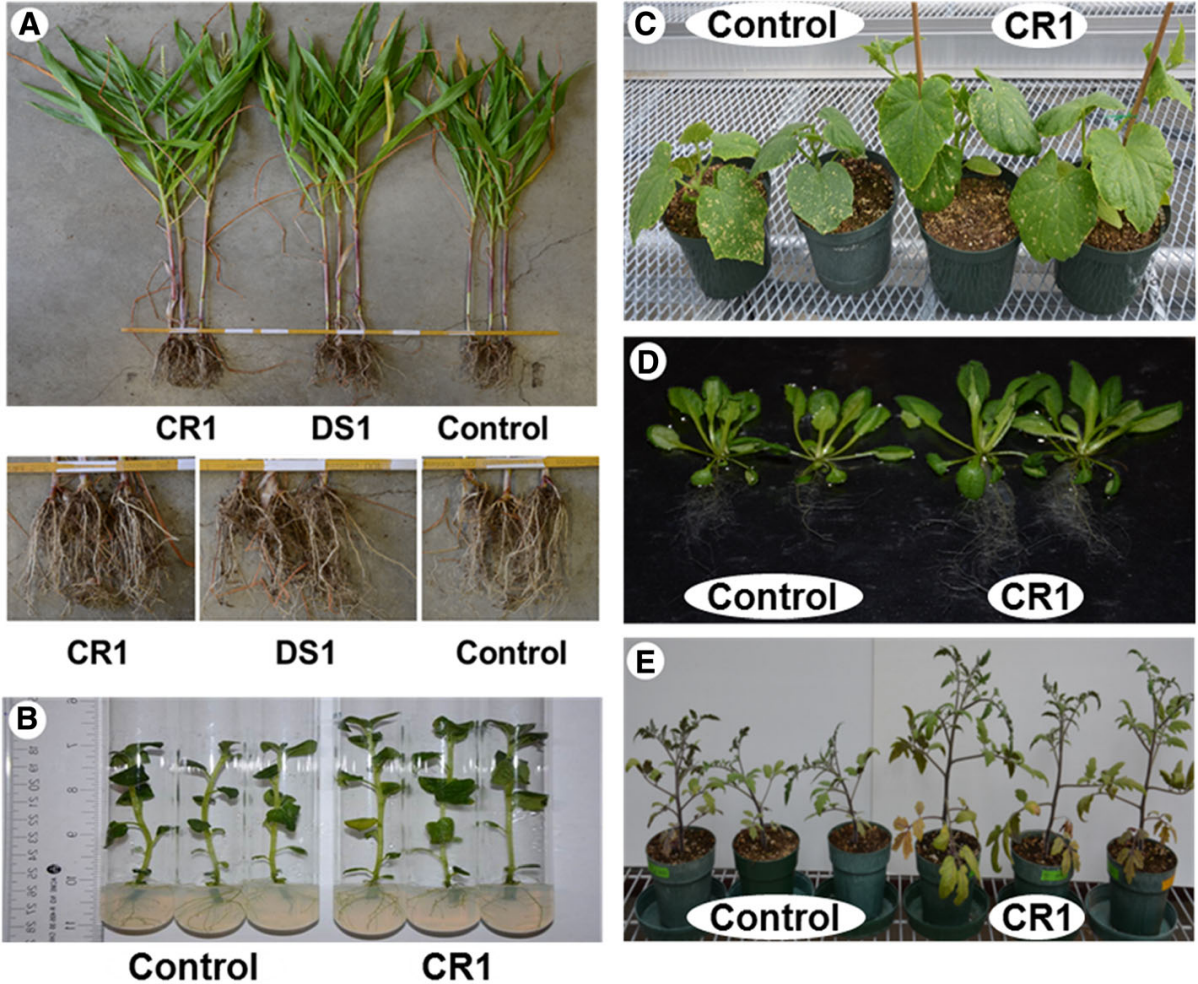
Plant growth promotion by *P. polymyxa* CR1. **a** Corn plants inoculated by seed soaking with *P. polymyxa* CR1 in reference to an established growth promoting rhizobacterium, *Gluconacetobacter azotocaptans* DS1 [30] and control plants. Bottom panel indicates increases density of root formation. **b** Tissue-culture potato plantlets grown as single-node explants following inoculation with *P. polymyxa* CR1 culture, and control plantlets. **c** Cucumber plants inoculated at the stem base with *P. polymyxa* CR1, and control plants. **d** *A. thaliana* Col-0 inoculated at the stem base with *P. polymyxa* CR1, and control plants. **e** Tomato plants inoculated at the stem base with *P. polymyxa* CR1, and control plants

Inoculation of plants with *P. polymyxa* CR1 resulted in increased plant growth relative to controls (Fig. 3). Inoculation of maize lead to a 19 % ± 2.3 % SD increase in whole-plant dry weight versus the mock-inoculated control after 8 weeks. In comparison, maize plants inoculated with *Gluconacetobacter azotocaptans* DS1, an established growth-promoting rhizobacterium [30], lead to a 13 % ± 1.2 % SD increase in dry weight versus the control. Inoculation with either bacterium appeared to enhance development of maize roots (Fig. 3a). Potato plantlets inoculated with *P. polymyxa* CR1 were 16 % ± 1.7 % SD taller than control plantlets after four weeks (Fig. 3b). Inoculation with *P. polymyxa* CR1 led to a 27 % ± 2.1 % SD increase in cucumber shoot fresh weight after four weeks (Fig. 3c), a 25 % ± 3.1 % SD increase in *Arabidopsis thaliana* Col-0 dry weight after three weeks (Fig. 3d), and a 27 % ± 2.3 % SD increase in tomato shoot dry weight after six weeks (Fig. 3e), relative to the controls.

Beneficial rhizobacteria can promote plant growth through bacterial nitrogen fixation, IAA hormone production, phosphorus solubilization, or by protecting plants from phytopathogens [4], such as the ones studied above. Our experiments with maize, cucumber, and tomato were conducted in non-sterile soil in a greenhouse. However, the experiments with potato and *A. thaliana* were in soil-free media with agar. While these latter results may not be representative of effects in nature (soil), they do provide evidence of growth promotion due to direct effects of P. polymyxa CR1 inoculation, rather than indirect effects on other rhizosphere microbes. Therefore, factors other than pathogen antagonism, such as IAA production, phosphate solubilization and nitrogen fixation, were expected to have collectively contributed to the observed growth promotion.

### Nitrogen fixation

**Fig. 4:**
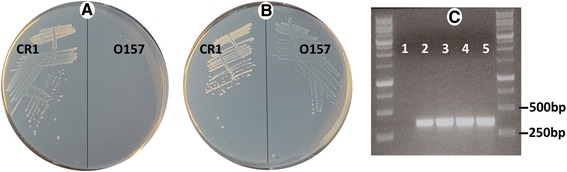
Nitrogen-fixation by *P. polymyxa* CR1. *P. polymyxa* CR1 inoculated on nitrogen free media (NFM) was assessed for growth in reference to non-nitrogen fixing *E. coli* O157:H7. **a** NFM medium; **b** NFM supplemented with 5 mM NH_4_Cl; **c** The presence of nitrogen fixing genes. Primer pairs PolF and PolR were utilized to amplify 352 bp of the *nifH* gene from four single colonies of the same *P. polymyxa* CR1 strain (lanes 2–5), or in a PCR reaction control without DNA (lane 1). Presence of a *nif* gene cluster was confirmed by DNA sequencing

The vegetative growth of plants (leaves, stems, and roots) is especially dependent on nitrogen fertilizer [1]. The nitrogen-fixing potential of *P. polymyxa* CR1 was therefore assessed by growth on nitrogen-free minimal medium (NFM). Growth of *P. polymyxa* CR1 was visible on the NFM, while *Escherichia coli* O157:H7, which does not fix nitrogen, grew only on medium supplemented with reactive nitrogen (Fig. 4). These results demonstrate the ability of *P. polymyxa* CR1 to fix nitrogen, which is not surprising given the presence in the *P. polymyxa* CR1 genome of the *nif* gene cluster [20] that is sufficient to confer nitrogen fixation to *Escherichia coli* [30].

Nitrogen fertilizers are routinely applied to crops to ensure growth and productivity. However, more than half of synthetic nitrogen fertilizer is not taken up by crops, instead being lost to the environment where it contributes to greenhouse gas production, acid rain, and biodiversity loss in aquatic systems [31]. These detrimental effects could be lessened by inoculating fields with nitrogen-fixing rhizobacteria, allowing for partial replacement of synthetic fertilizers. Accordingly, *P. polymyxa* CR1 may be able to contribute to the bio-fertilization of a variety of crops.

### Phosphorus solubilization

Like nitrogen, phosphorus is another major essential macronutrient for plant growth, health and productivity [1]. Phosphate based fertilizers (mineral phosphate) are routinely applied to soil in agricultural practice. However, applied phosphate fertilizer is rapidly immobilized through precipitation reaction with highly reactive Fe3+, aluminum and calcium in soil and consequently becomes unavailable to plants [7, 32]. Soil erosion also leads to phosphorus loss from soils, which usually accumulates in lakes and rivers causing eutrophication. Microbe-mediated phosphorus solubilization is one of the most important traits relevant to plant growth promotion [7, 32–34].

**Fig. 5:**
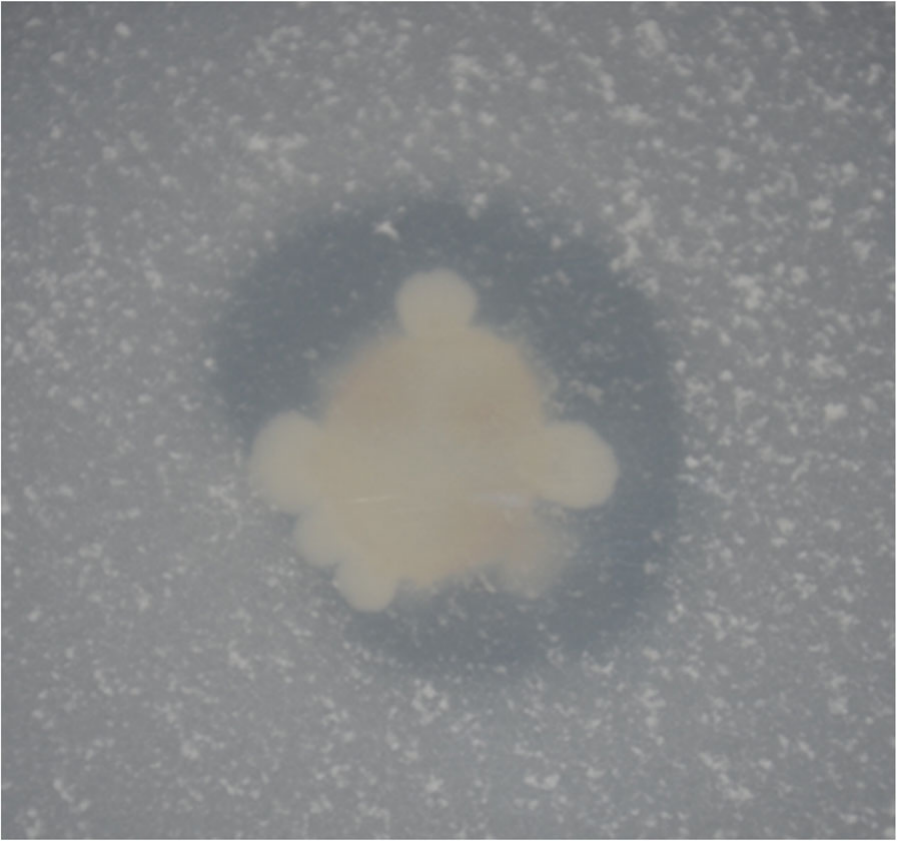
Inorganic phosphate solubilization by *P. polymyxa* CR1. CR1 was immobilized on NBRIP plates as described in materials and methods. CR1’s capacity to solubilize and use Ca_3_(PO_4_)_2_ as a sole source of phosphate was evident as a clearing zone around CR1 inoculum

*P. polymyxa* CR1 was assessed for its ability to solubilize and utilize inorganic phosphate in the form of tricalcium phosphate [Ca_3_(PO_4_)_2_]. After two weeks of incubation, clear and visible dissolution halos formed around a *P. polymyxa* CR1 colony grown on solid NBRIP medium, in which Ca_3_(PO_4_)_2_ is the sole phosphate source (Fig. 5). These dissolution halos indicate the excretion of organic acids or enzymes into the surrounding medium that solubilize Ca_3_(PO_4_)_2_. The results therefore suggest an ability to solubilize inorganic phosphate to a form available for plants, although they do not necessarily imply phosphate solubilisation in nature.

### Indole 3 acetic acid (IAA) production

Another way that rhizobacteria can promote plant growth is through synthesis of the phytohormone IAA [35–37], which plays a multifaceted role in plant growth and development. IAA stimulates plant cell elongation and cell division, increasing root size and distribution which leads to greater nutrient absorption from the soil [38, 39]. Appropriate concentrations of exogenous IAA or IAA agonists also stimulate the growth and development of plant root systems by activating root branching and lateral root development [10, 11].

The ability of *P. polymyxa* CR1 to synthesize IAA was determined by reaction of liquid culture with Salkowski’s reagent, which detects IAA and its intermediates [9]. The reaction produces a proportionate amount of tric-(indole-3-acetato) iron (III) complex, which is pink in color and can be quantified by measuring absorbance at 535 nm. *P. polymyxa* CR1 produced an average of 62.8 μg/mL ± 3.4 μg/mL SD of IAA (or its intermediates) when grown with 500 mg/L L-Tryptophan (the precursor for IAA biosynthesis) in liquid MGN or NPT medium (Table 1). The difference in IAA production between MGN and NPT medium, with or without tryptophan, was not significant. The genomes of many plant growth promoting bacteria, including *P. polymyxa* [19], encode a transaminase for the oxidative deamination of L-tryptophan to yield indole-3-pyruvic acid as an intermediate for IAA production. As expected, significantly more IAA (or its intermediates) was produced in media with tryptophan than without (*p* < 0.01), indicating that *P. polymyxa* CR1 utilizes this tryptophan-dependent pathway.

**Table 1:** Indole-3-acetic-acid (IAA) production by *P. polymyxa* CR1 grown in various media

	IAA concentrations (μg/mL) in replicate cultures			
	WGN	WGN + Trp	NPT	NPT + Trp
replicate #1	0.9	67.1	4.4	64.2
replicate #2	0.2	65.0	2.4	64.1
replicate #3	0.0	57.8	1.9	58.9

### Degradation and utilization of lignin, cellulose and hemi-cellulose

**Fig. 6:**
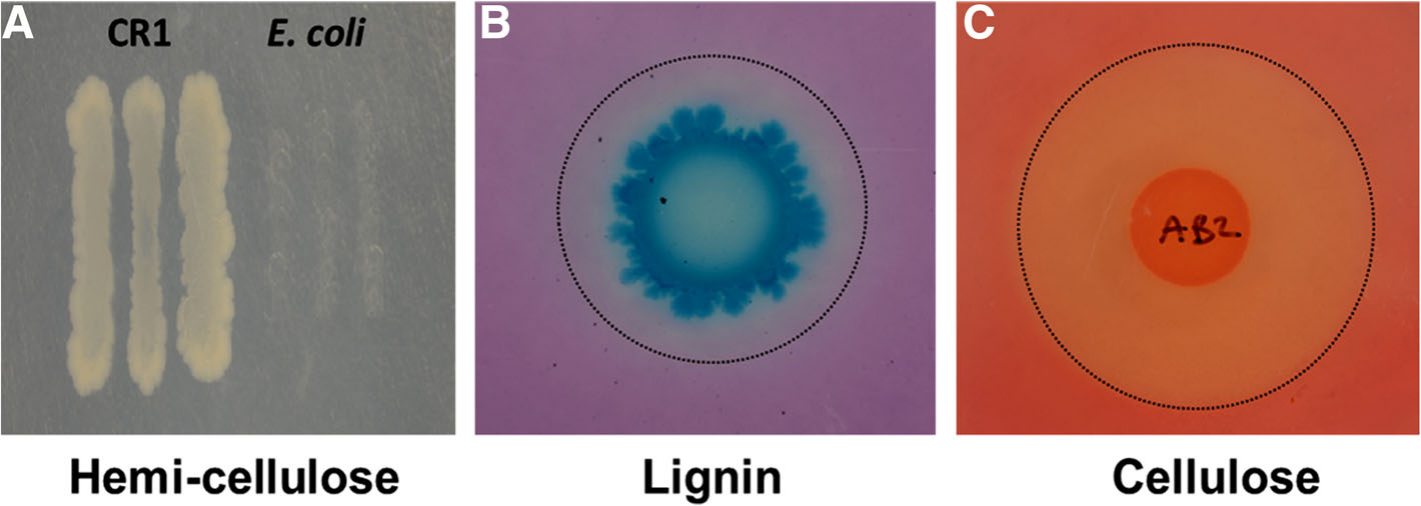
*P. polymyxa* CR1 degrades and utilizes semi-cellulose, lignin and cellulose as a sole carbon sources. **a**) CR1 (left) inoculum on minimal media supplemented with hemi-cellulose compared against *E. coli* O157:H7 (right). **b**) CR1 ligninolytic enzyme activity detected by halo surrounding inoculum on minimal medium containing methylene blue. **c**) Congo red staining for cellulose activity

In addition to its growth promoting properties, *P. polymyxa* is known to produce a variety of lignocellulose-modifying enzymes [13] which can be used in a variety of applications. The ability of *P. polymyxa* CR1 to utilize hemicellulose was confirmed by growth on minimal medium supplemented with xylan (Fig. 6a). *P. polymyxa* CR1 was grown on minimal medium supplemented with methylene blue, a lignin mimetic, to detect ligninolytic enzyme activity [40]. The oxidation zone (clear halo) around the colony (Fig. 6b) is indicative of lignin metabolism. Cellulose degradation was confirmed by cultivation with carboxymethylcellulose and Congo red, which stains the un-degraded substrate [41], producing a concentric yellow opaque zone of clearing around the colony (Fig. 6c). Our recent genome sequencing confirmed the presence of genes encoding extensive plant cell wall-degrading machinery in the CR1 genome including endoglucanases, cellodextrinases, xylanases, mannanases, arabinofuranosidase, DyP-peroxidase, and laccase [20].

Lignocellulose-degrading bacteria can contribute to nutrient cycling in untilled land by promoting biological decomposition of residues from non-harvested plant parts. Lignocellulose-modifying *P. polymyxa* CR1, or its isolated enzymes, could also be studied for their potential use in biomass delignification or carbohydrate deconstruction by the biofuels industry. As part of the process for conversion of lignocellulose to fuel or value-added bio-products, it could contribute to a reduced dependency on petrochemicals [42]. To better assess this potential, enzymatic activities (cellulases, xylanases, lignin peroxidases and laccases) will need to be quantitatively assessed under industrially-relevant conditions, and if justified, optimal fermentation conditions need to be investigated.

## Conclusions

*P. polymyxa* CR1 possesses several beneficial properties and enhances growth of a variety of important plants. Like most studied strains of *P. polymyxa*, it has the ability to produce the beneficial plant hormone IAA and to solubilize inorganic phosphorous. It is also able to fix atmospheric nitrogen, a trait that is shared by only a select few of the species. Furthermore, while each strain presumably antagonizes a unique set of microorganisms, *P. polymyxa* CR1 showed antagonistic activity toward key plant pathogens that are phylogenetically diverse. In addition, the strain was shown to utilize and degrade the main components of lignocellulose. To our knowledge, this is the first time that a single *P. polymyxa* strain has demonstrated all of these advantageous traits.

Due to the multiple beneficial effects of *P. polymyxa* CR1, this strain could be developed and commercially formulated, either alone or as part of microbial consortia, for field application in order to control pathogens, promote crop growth, and degrade crop residues after harvest. Further research is needed to establish optimum growth parameters of *P. polymyxa* CR1, determine factors influencing its competitiveness in soil, and confirm these benefits in field trials.

## Methods

### 16S rRNA sequencing and phylogenetic tree construction

Approximately 1,500 bp of the 16S rRNA gene were amplified from *P. polymyxa* CR1 genomic DNA isolated using the Bacterial Genomic DNA Isolation Kit (Norgen Biotek Corp., Thorold, ON, Canada) in accordance with the manufacturer’s protocols. The PCR reaction was performed with 2.5 μM each primers 8 F (5′-AGAGTTTGATCCTGGCTCAG-3′) and 1492R (5′-GGTTACCTTGTTACGACTT-3′) [43], 1.5 U Phusion High-Fidelity DNA Polymerase (Thermo Fisher Scientific Inc., Waltham, MA, USA), 50 ng genomic DNA, 200 μM dNTPs, 1X PCR buffer and 2.0 mM MgCl2 in a total volume of 50 μL. The cycle parameters were as follows: initial denaturation at 95 °C for 5 min; 30 cycles of denaturation for 30 s at 94 °C, annealing for 45 s at 57 °C, and extension for 60 s at 72 °C; and a final overall extension for 10 min at 72 °C. The PCR product was purified using the QIAquick PCR Purification kit (Qiagen, Hilden, Germany) and sequenced with 8 F and 1492R primers on a 3730 Analyzer (Thermo Fisher Scientific Inc.) at Agriculture and Agri-Food Canada (London, ON, Canada).

The 16S rRNA gene fragment was compared with the NCBI nucleotide database using Blastn. The following bacterial 16S rDNA from taxonomically characterized homologues were collected from the Genbank database on NCBI (http://www.ncbi.nlm.nih.gov/genbank) and used for phylogenetic analysis: *Paenibacillus polymyxa* M1 (HE577054.1)*, Paenibacillus polymyxa* SC2 (CP002213.1)*, Paenibacillus polymyxa* ATCC 842 (AFOX01000032.1)*, Paenibacillus polymyxa* E681 (CP000154.1)*, Paenibacillus terrae* HPL-003 (NR_075028.1)*, Paenibacillus favisporus* GMP01 (NR_029071.1)*, Paenibacillus dendritiformis* NB12 (JN215506.1)*, Streptococcus pneumoniae* ATCC 33400 (NR_028665.1)*, Bacillus subtilis subsp. subtilis* 6051 (NC_020507.1)*, Listeria monocytogenes* NCTC10357 (NR_044823.1)*, Clostridium septicum Pasteur III* (NR_026020.1)*, Acidimicrobium ferooxidans* DSM 10331 (NR_074390.1)*, Steptomyces coelicolor* QU66C (AB588124.1)*, Corynebacterium diphtheriae* VA01 (NR_103937.1)*, Mycobacterium leprae* Br4923 (NR_102782.1)*, Mycobacterium tuberculosis* NCTC 7416 H37Rv (NR_044826.1)*.* Multiple alignments were performed using the CLUSTAL_X program [44] and manually refined. A phylogenetic tree based on a comparison of 1500 bases was constructed using the neighbour-joining [45] and maximum-parsimony [46] methods using the software package MEGA version 4.1 [47]. Evolutionary distances were calculated according to Kimura’s two-parameter model [48]. Alignment gaps and ambiguous bases were excluded from the calculation. The topology of the tree was evaluated by bootstraping with 1000 pseudo-replicates. Similarity values were calculated using paup, version 4.0b1 [49].

### Isolation of bacteria from degrading corn roots

Degrading corn roots were collected from the research farm at London Research and Development Centre, Agriculture and Agri-Food Canada, in London, Ontario. Forty grams of degrading corn roots were first rinsed with water followed by 10 thorough washes with autoclaved distilled water to remove adhering soils and microbes that were not tightly associated with roots (no surface sterilization). For the preparation of root bacterial suspensions, root samples were cut into 1-5 mm lengths, suspended in 10 mL sterile 0.85 % NaCl and homogenized with a sterile mortar and pestle. Resulting bacterial suspensions were serially diluted with 1 mL of sterile 0.85 % NaCl to a final dilution of 10^-9^. Subsequently, 100 μL aliquots were plated onto 1/5 NPT (0.4 g/L nutrient broth, 1 g/L potato dextrose, 1.2 g/L tryptic soy broth, 2 g/L MES hydrate, 15 g/L agar, pH 5.75) and incubated at 28 °C for 72 h. Individual bacterial colonies were isolated and colony purified twice prior to in vitro antagonism assays.

### In vitro antagonism assay

Dual culture assays tested the antagonistic abilities of isolated bacteria against six microorganism. *P. sojae* P6497, *R. solani* 1809, and *C. destructans* 2062 were routinely cultured in potato dextrose agar (PDA) at 25 °C, and working stocks were established by transferring a cultured agar plug onto solid 1/5 NPT at 25 °C for 7 days. *P. syringae* DC3000, *X. campestris* 93-1, *B. cereus* BcMOR28, and the isolated bacterial strains were maintained in 1/5 NPT. Purified isolated bacteria were frozen in 15 % w/v glycerol at -80 °C for long-term storage.

Anti-microbial activity of selected isolates was assessed under in vitro conditions. Bacterial isolates were cultured on 1/5 NPT agar overnight at 28 °C. Pathogenic bacteria were spread as a lawn on separate 1/5 NPT agar plates and allowed to air-dry in a laminar-flow cabinet. Then, 10 μL aliquots of isolated bacterial suspensions (10^8^ CFU/mL) pre-grown in 1/5 NPT were evenly spotted on the pathogenic bacterial lawn, with eight different bacterial isolate spots per lawn. The plates were then incubated at 28 °C for three days, by which time a visible zone of inhibition, which is clear of pathogenic bacterial growth, surrounded the spotted bacterial isolates on some of the plates. A zone of inhibition can form due to diffusion of bacterial metabolites into the agar [50].

For antifungal and anti-oomycete activity, mycelial plugs of 1 cm diameter were cut from a 6-day-old culture and transferred to the center of a Petri dish containing 1X NPT medium. After 24 h incubation at 25 °C, bacterial isolates were streaked in a line approximately 2 to 3 cm away from the mycelial plug. The plates were then incubated at room temperature (approximately 25 °C) for 10 days, until radial growth of the mycelia reached the edge of the plate on the side without bacterial inoculation.

Bacterial isolates that demonstrated consistent antagonistic activity on three replicate plates were single colony purified three times prior to further study or storage.

### Plant growth promotion experiment

*P. polymyxa* CR1 grown in 1/5 NPT and 28 °C for 24 h were harvested and diluted in distilled water or nutrient broth for inoculation of plants. Approximate colony forming units per milliliter (CFU/mL) were determined by optical density and serial dilutions with plate counts.

Tissue-cultured potato plantlets (*Solanum tuberosum* L. cv. Kennebec) derived from stock plantlets (New Liskeards Agricultural Research Station, New Liskeard, Ontario, Canada) were grown from 1 cm long single-node explants in 22 x150 mm test tubes containing 10 mL MS -based potato nodal cutting medium (PNCM: 4.4 g/L MS basal salt medium (Sigma-Aldrich Corp., St. Louis, MO, USA), 30 g/L sucrose, 8 g/L noble agar, pH 6.0). The single-node explants were immersed in 10^9^ CFU/mL bacterial suspension in distilled water, or distilled water only. Explants were then rinsed in sterile distilled water for 1 min, and air dried on a sterile paper towel prior to planting in PNCM. Tubes were wrapped with 1 in. Micropore tape (3 M, Maplewood, MN, USA) to allow air flow to the plants while preventing fungal spores from entering the tube. Plants were grown in a controlled environmental chamber with an 18 h / 6 h light / dark cycle with temperatures at 22 °C (light) and 18 °C (dark).

Wild-type (WT) *Arabidopsis thaliana* Col-0 seed (stock no. CS70000) were ordered from the Arabidopsis Biological Resource Center website (https://abrc.osu.edu/order-stocks). Seeds were surface sterilized with 5 % sodium hypochlorite (100 % commercial laundry bleach), rinsed five times with sterile water, and kept at 4 °C in the absence of light for 2 days to stimulate germination. Seeds were then sown on square Petri dishes (100 × 100 × 15 mm) with half strength semi-solid Murashige and Skoog medium (MS: half packet/L MS Basal salts, 7.5 g/L sucrose, 0.25 g/L MES powder, 59 mL/L B5 vitamin mix, 3.5 g/L agar, pH of 5.75 using NaOH) and placed in a growth room with a 14 h / 10 h light / dark cycle with a total light intensity of 200 μmol photons m − 2 s − 1, temperature of 21 ± 4 °C, and 40 ± 10 % relative humidity. After two days, the plants were inoculated with 50 μL of 10^9^ CFU/mL bacterial suspension in water, or distilled water only, by streaking below the region of the planted seed.

For tomato, cucumber and corn, seeds were soaked in 10^8^ CFU/mL bacterial suspension in nutrient broth, or nutrient both only, before sowing into soil and grown in a greenhouse. Soil consisted of equal amounts of Pro-Mix BX (Premier Tech Horticulture, Rivière-du-Loup, QC, Canada) and native soil obtained on-site from the London Research and Development Centre, Agriculture and Agri-Food Canada. After 7 days, 300 μL of 10^8^ CFU/mL bacterial suspension in nutrient broth, or nutrient broth only, were inoculated at the base of the plant stems near the substrate interface.

### Nitrogen fixation assay

A single colony of *P. polymyxa* CR1 or *Escherichia coli* O157:H7 grown on solid 1/5 NPT medium was streaked onto solid nitrogen-deficient malate medium (NFM: 0.1 g /L NaCl, 0.02 g/L CaCl_2_, 0.4 g/L KH_2_PO_4_, 0.5 g/L K_2_HPO_4_, 0.2 g/L MgSO_4_.7H_2_O, 0.01 g/L FeCl_3_, 0.002 g/L Na_2_MoO_4_.2H_2_O, 5 g/ L sodium malate, 15 g/L agar, pH 7.2-7.4 using KOH) supplemented with 50 mg/L yeast extract [51]. A resulting single colony was then re-streaked onto NFM to confirm the ability to fix nitrogen [52]. Plates were incubated at 28 °C for 7 days.

### Phosphate solubilization assay

Phosphate solubilization was evaluated as previously described [53]. Briefly, *P. polymyxa* CR1 was cultivated on solid NBRIP medium (1 % glucose, 0.5 % Ca_3_(PO_4_)_2_, 0.5 % MgCl_2_, 0.01 % (NH_4_)_2_SO_4_, 0.025 % MgSO_4_.7H_2_O, 0.02 % KCl, 1.5 % agar), where growth is associated with the capacity to use inorganic phosphate in the form of Ca_3_(PO_4_)_2_ as a sole phosphate source. Plates were grown at 28 °C for 14 days.

### Indole 3 acetic acid (IAA) production assay

Production of IAA and related compounds was based on the colorimetric method previously described, with some modifications [9, 54, 10]. A 20 μL aliquot of bacterial culture adjusted to an OD_600_ of 0.5 (10^6^ – 10^7^ CFU/mL) was used to inoculate 3 mL of liquid MGN (10 g/L mannitol, 2.32 g/L sodium glutamate, 0.5 g/L KH_2_PO_4_, 0.2 g/L MgSO_4_, 0.2 g/L NaCl, 2 μg/L biotin, 1 g/L nutrient broth, 2.5 g/L potato dextrose, 3 g/L tryptic soy broth, 1 g/L MES hydrate, pH 6) or NPT (2 g/L nutrient broth, 5 g/L potato dextrose, 6 g/L tryptic soy broth, 2 g/L MES hydrate, pH 5.75), with or without 500 mg/L L-Tryptophan as the precursor for IAA biosynthesis. After 42 h incubation at 28 °C and 150 rpm, the culture was centrifuged at 5,500 x g for 10 min, and 1 mL of supernatant was mixed with 4 mL of Salkowski’s reagent (150 mL concentrated H_2_SO_4_, 250 mL distilled H_2_O, 7.5 mL 0.5 M FeCl_3_•6H_2_O) by vortexing briefly. The mixture was incubated in the dark at room temperature for 20 min [54] and absorbance was measured at 535 nm using a SmartSpec Plus Spectrophotometer (Bio-Rad Laboratories Inc., Hercules, CA, USA). Equivalent mixtures with non-inoculated media served as blanks for the spectrophotometry readings. IAA concentrations were determined using a standard curve for each medium type (MGN and NPT, with or without 500 mg/L L-Tryptophan) spiked with 0 to 400 μg/mL crystalline IAA (Sigma-Aldrich Corp.). Values presented were divided by 5, as the culture was diluted 5 x in Salkowski’s reagent.

### Lignin, cellulose, and hemicellulose degradation assays

Isolated bacterial colonies were transferred to minimal media (6 g/L NaCl, 1 g/L (NH_4_)_2_SO_4_, 0.5 g/L KH_2_PO_4_, 0.5 g/L K_2_HPO_4_, 0.1 g/L MgSO_4_, 0.1 g/L CaCl_2_, 2 g/L MES, 15 g/L agar, pH 5.75 using NaOH) supplemented with 1 g/L of either alkali lignin, colloidal microcrystalline cellulose, or birchwood xylan (all from Sigma-Aldrich Corp.) as sole carbon sources. Plates were incubated at 37 °C for 4 days, and bacteria were transferred to fresh plates in order to confirm the results.

Cellulose degradation was detected by transferring colonies to minimal medium containing 1 % w/v carboxymethylcellulose and 0.1 w/v glucose. After 48 h, the plate was flooded with 5 mL of 0.1 % w/v Congo red (Ricca Chemical Co., Arlington, TX, USA) and stained for 15 min at room temperature. After removing the dye, the plate was washed twice with 5 mL 1 M NaCl for 15 min each, then air dried and photographed.

Ligninolytic enzyme production was detected by transferring colonies to minimal medium containing 25 mg/L methylene blue at 28 °C for 72 h.

## Abbreviations

ACC: 1-aminocyclopropane-1-carboxylate
IAA: indole-3-acetic acid
CFU: colony forming units
CR1: corn rhizobacterium 1
MES: 2-(N-morpholino)ethanesulfonic acid
MGN: medium containing mannitol, glutamate, nutrient broth, potato dextrose, tryptic soy, KH_2_PO_4_, MgSO_4_, NaCl, biotin, and MES
MS: Murashige and Skoog medium
NBRIP: medium containing glucose, Ca_3_(PO_4_)_2_, MgCl_2_, (NH_4_)_2_SO_4_, MgSO_4_, and KCl. Growth is associated with the capacity to use inorganic phosphate in the form of Ca_3_(PO_4_)_2_ as a sole phosphate source
NFM: nitrogen-free minimal medium
NPT: medium containing nutrient broth, potato dextrose, tryptic soy, and MES
PDA: potato dextrose agar
PNCM: potato nodal cutting medium
WT: wild-type
